# DEMENTIA DIAGNOSIS, HEALTHCARE USE, AND OUTMIGRATION OF OLDER ADULTS IN PUERTO RICO AFTER HURRICANE MARIA

**DOI:** 10.1093/geroni/igae098.4156

**Published:** 2024-12-31

**Authors:** Jeung Hyun Kim, Yoojin Lee, Yanru Liao, Maricruz Rivera-Hernandez

**Affiliations:** Brown University, Providence, Rhode Island, United States; Brown University, Providence, Rhode Island, United States; Center for Gerontology and Healthcare Research, Brown University, Rhode Island, United States; Brown University School of Public Health, Providence, Rhode Island, United States

## Abstract

Puerto Rico’s historic net migration loss spiked after Hurricane Maria, which devastated its economy and health system (e.g., from 1.4% to 3.4% outmigration among all Puerto Rican Medicare beneficiaries and about 60% increase in the net-migration loss before/after September 2017). This research examined whether the diagnosis of Alzheimer’s Disease and Related Dementias (ADRD) among the older adults in Puerto Rico associated with their decision to migrate to the U.S. mainland, particularly after the year of the Hurricane. The data was derived from the Master Beneficiary Summary File (MBSF), and focused on fee-for-service enrollees in Puerto Rico (n=72,117 from the MBSF 2017/67,985 from the MBSF 2018). Logistic regression showed that older adults who were diagnosed with ADRD were significantly more likely to move to the mainland compared to the non-ADRD individuals in 2017 and 2018. In addition, higher levels of healthcare utilization were strongly related to the outmigration decision. When stratified by age (i.e., 65+ or 80+), these associations remained consistent. These results may suggest that older adults with ADRD in Puerto Rico may have moved to the mainland to seek better healthcare (i.e., long-term care) in the years after the Hurricane, and higher levels of their healthcare use may have contributed to their outmigration decisions. While many studies focus on the young generation to capture the Hispanic migration flow to the U.S., our study shows that the complex health needs of older adults, including the very old ones, propel the emerging outmigration trend in Puerto Rico.

